# Feasibility of extracting data from electronic medical records for research: an international comparative study

**DOI:** 10.1186/s12911-016-0332-1

**Published:** 2016-07-13

**Authors:** Michelle Helena van Velthoven, Nikolaos Mastellos, Azeem Majeed, John O’Donoghue, Josip Car

**Affiliations:** Global eHealth Unit, Department of Primary Care and Public Health, Imperial College London, London, UK; Lee Kong Chian School of Medicine, Imperial College & Nanyang Technological University, Singapore, Singapore

**Keywords:** Electronic medical records, Electronic health records [MeSH], Data collection [MeSH], Global health [MeSH]

## Abstract

**Background:**

Electronic medical records (EMR) offer a major potential for secondary use of data for research which can improve the safety, quality and efficiency of healthcare. They also enable the measurement of disease burden at the population level. However, the extent to which this is feasible in different countries is not well known. This study aimed to: 1) assess information governance procedures for extracting data from EMR in 16 countries; and 2) explore the extent of EMR adoption and the quality and consistency of EMR data in 7 countries, using management of diabetes type 2 patients as an exemplar.

**Methods:**

We included 16 countries from Australia, Asia, the Middle East, and Europe to the Americas. We undertook a multi-method approach including both an online literature review and structured interviews with 59 stakeholders, including 25 physicians, 23 academics, 7 EMR providers, and 4 information commissioners. Data were analysed and synthesised thematically considering the most relevant issues.

**Results:**

We found that procedures for information governance, levels of adoption and data quality varied across the countries studied. The required time and ease of obtaining approval also varies widely. While some countries seem ready for secondary uses of data from EMR, in other countries several barriers were found, including limited experience with using EMR data for research, lack of standard policies and procedures, bureaucracy, confidentiality, data security concerns, technical issues and costs.

**Conclusions:**

This is the first international comparative study to shed light on the feasibility of extracting EMR data across a number of countries. The study will inform future discussions and development of policies that aim to accelerate the adoption of EMR systems in high and middle income countries and seize the rich potential for secondary use of data arising from the use of EMR solutions.

**Electronic supplementary material:**

The online version of this article (doi:10.1186/s12911-016-0332-1) contains supplementary material, which is available to authorized users.

## Background

### Characteristics and benefits of electronic medical records (EMR)

Electronic medical records (EMR) offer a major potential to improve the safety, quality and efficiency of healthcare [1]. The International Organisation for Standardization defines EMR (often referred to as electronic health or patient records, computerised medical or patient records and Electronic Health Record) as a “repository of information regarding the health status of a subject of care, in computer processable form” [2]. Thus, EMRs are an electronic version of patients’ health records which can be used for input, storage, display, retrieval and sharing of information [3]. Accurate and complete data from EMRs can be used by practitioners to improve the safety, quality and efficiency of care. For example, EMRs have been used to provide physicians with data regarding type 2 diabetic patients, which has shown to improve process measures such as an increased number of foot and eye check-ups and biological outcomes including glycated haemoglobin (known as “HbA1c”) and blood glucose [4].

There are several secondary uses of data from EMR that can improve healthcare services, including population and disease research, detection of adverse drug reactions, assessment of outcomes of interventions, shaping health policies, and guidance on effective use of resources [5–7]. EMR data can be an indispensable source for population and disease research especially when it can be linked with mortality records and genetic data. Data can be made available for research through different mechanisms; for example in the United Kingdom, large primary care databases, the Clinical Practice Research Datalink system [8], the Health Improvement Network [9] and QResearch [10], provide access to National Health Service observational data and interventional research. This data is used for various areas of research including, cardiovascular disease, mental health and pharmacoepidemiology [9]. EMR data are also invaluable to the pharmaceutical industry which, for example, can use data to improve the safety of medication use by monitoring side-effects and interactions with other medication [11]. A major potential benefit of secondary data analysis of EMR data is its use to improve global burden of disease measurement, especially, though not exclusively, of non-communicable diseases [12].

### Barriers to secondary use of EMR data

Despite the large investments in EMR systems worldwide, some countries have yet to realise the potential of EMR to improve the quality, safety and efficiency of healthcare [13, 14]. In many settings, electronically collected data is often not analysed at aggregate level, which limits our understanding regarding a population’s health needs, disease management, quality of management of chronic conditions and outcomes of interventions at both primary care and hospital settings [5]. There are several barriers constraining the potential of EMR data for secondary uses. An important barrier in some cases is the poor accuracy and completeness of EMR data [15, 16]. Other barriers include legal and ethical considerations required for secondary use of EMR data [7]. Information governance procedures can be a barrier as obtaining approvals to extract and analyse EMR data may be challenging given the novelty of this type of research in certain countries. Moreover, the diversity in EMR systems across different settings and differences in legal and information governance systems, social norms and frameworks pose additional challenges for obtaining approvals. Therefore, identifying and addressing current barriers towards secondary use of EMR data is of great importance to facilitate greater use of EMR solutions.

### State of EMR adoption internationally

Currently, there is limited evidence on the adoption of EMR internationally [5, 17]. Previous assessments have mainly focussed on the adoption of EMR systems in the United States and some other countries [4, 17–21]. A recent systematic review on the impact of EMR implementations found that nearly two-thirds of EMR studies took place in the United States (*n* = 62), a small number of studies were conducted in England and Denmark (*n* = 5), Canada (*n* = 3), and Norway (*n* = 4), and an even smaller number (*n* = 1–2) in other countries [17]. Moreover, according to the same review, there have been few international comparisons of EMR [17]; the review found only one paper comparing EMR implementations between countries (United States and Sweden) [22]. Given the novelty of using data from EMR in some countries, information regarding ethical and legal procedures that are required is also scarce.

### Aim of this study

This study aimed to assess information governance procedures for extracting data from EMR across 16 countries, using the management of type 2 diabetic patients as an exemplar. A secondary aim of the study was to explore the extent of EMR adoption and the quality and consistency of EMR data in 7 countries. Thereby, this study identifies barriers towards secondary uses of EMR data across different countries, which can be used to facilitate future analysis of EMR data.

## Methods

### Overview

This international comparative study covered 16 countries (Table 1). We assessed the adoption of EMR, quality of their data in 7 countries, Brazil, Italy, South Africa, Saudi Arabia, Korea, Rep., Taiwan and United Arab Emirates (UAE), and information governance processes for secondary uses of data in all 16 countries.

**Table 1:** The scope of assessment of countries included in this study

Country	Scope of assessment^a^	Region [49]	Income level [50]
Saudi Arabia	Full assessment	Eastern Mediterranean	High-income
United Arab Emirates (UAE)	Full assessment	Eastern Mediterranean	High-income
Taiwan	Full assessment	Western Pacific	High-income
Korea, Rep.	Full assessment	Western Pacific	High-income OECD
Italy	Full assessment	European	High-income OECD
South Africa	Full assessment	African	Upper-middle-income
Brazil	Full assessment	Americas	Upper-middle-income
Australia	Information governance	Western Pacific	High-income OECD
Austria	Information governance	European	High-income OECD
Czech Republic	Information governance	European	High-income OECD
The Netherlands	Information governance	European	High-income OECD
Poland	Information governance	European	High-income OECD
China	Information governance	Western Pacific	Upper-middle-income
Mexico	Information governance	Americas	Upper-middle-income
India	Information governance	South-East Asia	Lower-middle-income
Indonesia	Information governance	South-East Asia	Lower-middle-income

^a^Full assessment entails adoption of EMR, quality of their data and information governance processes

This study was commissioned by IMS Health (http://www.imshealth.com/) and they chose the countries and developed the questionnaires. The countries were selected for a planned non-interventional study, which included type 2 diabetes mellitus patients. The selection of countries was made based on preliminary expert advice and knowledge whether countries had a reasonable level of EMR adoption and limited fragmentation of EMR providers. The typical treatment settings for type 2 diabetes patients were general physicians and specialists in hospitals, but varied between countries (Additional files 1 and 2).

The study had a multi-method design which included peer-reviewed and grey literature review, email contact with relevant experts in countries studied and interviews with key stakeholders to collect information on governance processes, EMR adoption and data quality. We sought to identify the authorities and assess processes for approval of EMR data extraction and use for research, approximate time needed to obtain all approvals and expected ease of obtaining approval. We also examined the adoption of EMR systems within relevant treatment settings, EMR data quality, which was defined as typical fields covered, average fill rate (percentage of visits in which clinical information, including patient information, vitals, diagnosis, prescription, procedures, lab test results and patient behavioural, is being filled) and fields with close-ended questions, details of electronic health record systems, as well as trend and incentives on EMR use.

### Literature review

We conducted a literature review on academic papers using EMR data from type 2 diabetic patients, as well as other EMR extracted data (a description of the literature on adoption of EMR in the countries can be found in Additional file 3). Sources of information included scientific databases (e.g. PubMed and Google Scholar). Search terms included: electronic medical record*; Electronic Health Records [MeSH]; electronic health record*; electronic patient record*; computer-based or computerised medical record; computer-based or computerised health record; computer-based or computerised patient record; country name; and diabetes. Other optional terms included: adoption, uptake, coverage, governance, trend*, ethic*, and provider*. Where possible, we conducted more general literature searches (e.g. on Ministry of Health and EMR provider websites) to find additional information and contacts for the interviews. We included studies that reported on at least one of the following aspects of EMR: information governance procedures for extracting data from EMR; EMR adoption; and the quality and consistency of EMR data.

### Interviews

A structured interview questionnaire was used that included questions on: treatment setting of type 2 diabetes patients and EMR adoption within treatment settings; use of EMR data and existing relationships with EMR providers; information governance; trends and incentives on EMR use; and details of EMR systems. Contact details of interviewees were compiled through academic connections, contacting authors of literature review publications, searching the internet for research centres, hospitals, diabetes clinics and centres, specialists (diabetologists/endocrinologists) and contacting national and international diabetes organisations. Contacts were a mix of public and private sector professionals, including family practitioners and specialists with an emphasis on individuals in the field of diabetes, EMR providers, academics and information commissioners.

We requested interviews via phone and/or email. All interviewees were informed about the study and provided consent to participate (verbal for telephone calls and written for email). Five trained interviewers conducted phone interviews with participants from each country in English, apart from one interview that was conducted in Polish and transcribed verbatim and translated to English by a speaker native in both the English and Polish language. This was a rapid-response survey and due to time and/or language barriers, a small number of questionnaires were self-completed and sent via email. The number of conducted interviews ranged from 1 to 7 per country, depending on the availability of participants and the information required. We also engaged with those who provided information to self-assess reliability and comprehensiveness of information as an additional guide whether to seek further sources of information. We invited 377 informants and 59 participants were interviewed. Some participants were identified through expert referral and therefore we are unable to provide the exact number of those invited and those who declined participation. Of those who declined, some were unable to provide relevant information and others did not have time for participation in the study. Our purposive sample of 59 participants consisted of the following: 25 physicians, 23 academics, 7 EMR providers, and 4 information commissioners (Additional file 4). Interview data were analysed thematically. Two researchers read through the interview transcripts several times in an active way (searching for meaning) and gave initial codes to findings (units of texts). Then they searched for themes and sorted codes into themes. To verify the data, the results were shared with the research team and discussed.

### Data synthesis

We synthesised all information from the literature review documents and interview questionnaires thematically (Additional files 1 and 2). When large discrepancies in conflicting information (e.g. time to obtain approval) were found, we provided a range i.e. min to max. When only small discrepancies in conflicting information were found, we provided an average. Other conflicts of information were resolved by consensus among the team members, with a tendency towards the more trustworthy and competent source of information (assessment of those based on personal impressions).

## Results

### Information governance processes

#### Authorities and processes for approval for EMR data extraction and use for research

We obtained information regarding processes to obtain approval for EMR data extraction for research purposes in all countries apart from Austria where data from EMR systems were not yet meant to be used for research (Additional file 1). The procedures for obtaining approvals varied highly between countries. Approval processes varied significantly even between European countries. However, typically the different authorities from which approval had to be obtained to allow extraction of data from EMRs included ethics committees (health facility and/or regional or national boards), sites where data was collected, and national, regional or local health authorities. Additional approvals were needed from EMR providers in certain countries (Australia, Czech Republic, Italy, India, Poland and South Africa). There were also differences in approval procedures between different geographical areas within a number of countries (China [see information governance procedures described in Table 2], the Czech Republic, Indonesia, Italy, India, South Africa and UAE).

**Table 2:** EMR information governance: the example of China

Authorities who need to provide approval for EMR extraction and use of data for research purpose	• Ethics boards within the hospitals. • National and local health authorities e.g. Division of Medical Affairs and Division of Research: necessary if retrieving data from various provinces, or retrieving national data of various kinds such as public health, immunisation or epidemiology of emergency events. • Site where the data was collected: director of the hospital, hospital data centre. • Individual patients: In Hong Kong Special Administrative Region (HKSAR) consent needed for access to EHR. Not conclusive if required in mainland China. • In HKSAR: the Office of the Privacy Commissioner for Personal Data, an independent statutory body, oversees enforcement of the Personal Data Privacy Ordinance (PDPO). Although not applicable for anonymised data, users should comply with requirements under the PDPO personal data handling. Suspected breaches will be investigated.
Process to obtain approval	• Submission of Case Report Form (to ascertain potential harm to patients) and research proposal. • However, there is no clear legal framework about data use rights. • Process for Hong Kong: 1. Application to the Secretary for Food and Health; 2. The Secretary for Food and Health may refer application to the Electronic Health Record Research Board; 3. The Board must consider several factors including ethical issues and public interest; 4. Applications for non-identifiable data are made to the Commissioner for Electronic Health Records (eHRC).
Approximate time needed to obtain all approvals	Less than 3 months.
Ease of obtaining approval	Relatively easy, as there are procedures in place and the process is quick. The director of the hospital plays a key role in the approval process. Hurdles: • Organisation of the administration (no specific rules and regulations for data extraction). • Potential technical problems at some sites due to own systems in hospitals, and concerns of data leakage in China. • Law prohibiting transfer of non-anonymised EMR data outside HKSAR (section 33 of the PDPO), but not in force yet. • Anonymised patient data can be used for research and preparing statistics relevant to public health or public safety if conditions for approval by the future Commissioner for the EHR under Division 3 of the eHRSS Bill are fulfilled.
Regional differences	Regional differences exist, e.g. Hong Kong has own authority dedicated to data protection.

Additional approvals had to be obtained for data from the private sector in two countries (Australia, Poland). In addition, the approval procedures varied according to the type of study; for example, in Italy, participants said that a simple notification to the ethics board was needed for retrospective studies, while formal approval from the health authority was needed for prospective studies. Individual patient consent was often not required for anonymised data with the exception of South Africa where patient consent was always required. In some countries, obtaining patient consent was usual practice, though not necessary (Italy, the Netherlands).

#### Approximate time needed to obtain all approvals

The approximate time to obtain all the approvals that were required for extracting data from EMR highly varied. In 7 countries, the average time was between 3 and 6 months (Czech Republic, India, Indonesia, the Netherlands, Poland, Korea, Rep., UAE), whilst in 5 countries between 6 months and 1 year (Australia, Brazil, Italy, Mexico, Saudi Arabia). The time was expected to be less than 3 months for China, while variable times were reported for Taiwan (between 3 months and 6 years). In South Africa, an unsuccessful attempt for obtaining approvals lasted between 1 and 2 years. Typically the process could be lengthy and the time needed was dependent on the number of different sites that were to be included in a study.

#### Ease of obtaining approval

For most countries it was thought that obtaining approval was moderately easy. The exceptions were India and South Africa where this was difficult, as no standard procedures were in place to obtain approval, and Austria where obtaining approval was currently not feasible because of legal barriers.

The barriers to obtaining approvals were: (i) the novelty of using data from EMR for research, (ii) lack of standard procedures, (iii) bureaucracy, (iv) confidentiality, (v) technical issues and (vi) costs. Firstly, in some countries, there was little previous experience with conducting research using data from EMR (India, Indonesia, South Africa and Saudi Arabia). In another country, showing that a study had a real benefit to the health facility and public was reported to make approval procedures easier (Brazil). Secondly, a lack of uniformity of rules and regulations or policies for EMR data extraction was a challenge for obtaining approvals in three countries (China, India and South Africa). Sometimes, certain individuals had to be involved to obtain approval (South Africa, China); for example, one respondent said that in China the director of the hospital plays a key role in the approval procedure. Thirdly, the process was lengthier and more complex because of the need for approvals from: multiple organisations (Australia, Brazil, Korea, Rep., Taiwan, India), different levels within organisations (South Africa) and different stakeholders (India). One participant also mentioned that there were frequently delays in responses to approval requests (South Africa). Fourthly, there was a reluctance to share data because of concerns about confidentiality (Italy, Mexico), data security (Poland), and data leakage (China), which stressed the importance of developing trust to gain access to the data. Fifthly, technical issues, such as lack of interoperability (South Africa), limited bandwidth (UAE), difficulty with de-identification of data (Australia, Czech Republic) and identification of the correct EMR (India), were also mentioned as common barriers. These were challenging because there was a need for information technology specialists, but lack of experience and poor literacy among staff (South Africa). Finally, respondents considered the cost of administration (China), patents, utilisation and licensing (South Africa) and negotiating prices with EMR providers (Korea, Rep.) as additional barriers to obtaining approval.

### Adoption and quality of EMR

#### EMR adoption

EMR adoption was examined in 7 countries. Overall, Italy, Saudi Arabia, Korea, Rep., Taiwan and UAE had high EMR adoption rates while Brazil and South Africa had lower rates. However, EMR adoption rates were variable between different settings within countries (Additional file 2). In UAE, almost 100 % of Health Authority of Abu Dhabi (HAAD) affiliated healthcare facilities, known as Abu Dhabi Health Services Company (SEHA), had an EMR system. In Italy and Korea, Rep., around 90 % of general physician clinics used an EMR. However, in Italy, adoption rates varied between different regions and hospitals. In Korea, Rep., almost 80 % of tertiary hospitals and 40 % of smaller hospitals had an EMR. Finally, in Taiwan, all large medical centres (>1000 beds), approximately 70 % of regional hospitals and almost 30 % of district hospitals used an EMR.

#### The quality of EMR data and details on EMR systems

In six of these countries (excluding Brazil, see the full assessment described in Table 3), typical fields covered in EMR systems were: patient information, patient vital signs, diagnoses, prescriptions, information about hospitalisations, procedures/tests performed and lab test results. Average fill rates were reported to be high (75–100 %). The fields that were closed-ended were less clearly described and varied between settings. In Brazil, there was very little information regarding the fields and fill rates as this highly varied between clinics. Although it was possible to link information with systems from other sites in most countries (Italy, South Africa, Taiwan, UAE, Saudi Arabia), sometimes this was only for certain information, such as prescriptions, procedures performed and lab test results (Saudi Arabia).

**Table 3:** EMR adoption, data quality, implementation trends and incentives: the example of Brazil

Typical treatment settings for type 2 diabetes patients	• All basic care outside hospitals. • Public system is represented by public clinics (general physicians and specialists). Limited access to medication, usually low cost drugs given, dispensing often not recorded. • Private system (25 % of Brazilian population) consists of specialist physicians’ offices clinics. This is where most drug consumption takes place.
EMR adoption rate in the typical treatment setting	• Highly varied responses: general physicians 5–40 %, Specialists 5–50 %, Hospitals 7–80 %, Emergency units 50 %. Difficult to capture as it depends on each physician and office. • Low overall adoption. • Primary reason for reluctance to EMR and persistent paper culture: concerns among healthcare institutions regarding the security of patient data/concept of physicians owning their patient data and not wanting to forward this to other physicians.
Typical fields covered in the EMR system	• Depends on the type and structure of the system used by physician. This would be a clinic by clinic exercise. • No electronic prescriptions. • Both public and private hospitals (providing public services) have central database for claims data: only high-cost procedures and high-cost drugs dispensed. • More data captured in public system as attended by different physicians each time (EMR more favourable).
Average fill rate	Difficult to capture. This would be a clinic by clinic exercise.
Fields with close-ended questions	As physicians are protective of patient data, open fields may be more common than closed-ended. Some niche specialised systems exist with parameters used by the type of specialist, perhaps more likely to have close-ended questions.
Overall trend on EMR implementation	• Trend is growing and expanding slowly. • All initiatives are confined to private market. • No large changes in regulations or government mandatory imposed policy that will make change happen faster. • However, much interest from EMR vendors and accelerating activity expected in the next few years expected as Brazil is the third largest world market for EMR: with >200 million inhabitants, >7000 hospitals, >300,000 physicians, and a mixed public and private healthcare system. • The EMR market earned revenues of US $145 million in 2012 and estimates to reach US $336 million in 2018 at a compound annual growth rate of 15 percent.
Incentives for EMR implementation	• Most important incentive is the necessity to improve services and coordination of care as public and private health sectors are stretched. Likely to follow other countries in EMR implementation. • Incentives are sectorial, with independent motivations and initiatives. • Local healthcare information technology (HC IT) market stage drives providers to offer EMR as a module pack within a hospital information system (HIS) solution. • Very little national or regional incentives. In some states and some cities only.

#### Trend and incentives in EMR use

Overall, the trend for adoption of EMR was increasing in all 7 countries, but this was a slow process. Government incentives were poor in Brazil, but present in all other countries where EMR implementation was seen as a necessity to improve health services. However, EMR providers had a large interest to expand EMR adoption in Brazil’s large market.

## Discussion

This study adds important novel insights into the feasibility of extracting EMR data for research in 16 high– and middle-income countries. We assessed information governance processes and EMR adoption and quality in several countries for which no previous information in academic literature was available. We also examined different countries compared to previous EMR assessments [4, 17–21]. Thereby, this study identified barriers towards secondary uses of EMR data in different countries that can be used to inform future EMR data analysis.

We found that obtaining approval for extracting data from EMR for research was moderately easy in most of the 16 countries for which we assessed information governance procedures. Exceptions were India and South Africa where obtaining approval was difficult and Austria where obtaining approval was currently not feasible as data from EMR were not yet meant to be used for secondary purposes.

Our study found a mix of different levels of EMR adoption and data quality across the seven countries that were assessed. The five high-income countries achieved high adoption, while the two middle-income countries had lower EMR adoption rates. In Italy, Korea, Rep. and Taiwan, EMR implementation started more than 10 years ago and several governmental initiatives were in place to increase uptake. Also, research using EMR data was already published by the time of this study [23–28]. In the Middle-Eastern countries, Saudi Arabia and UAE, EMR research was relatively novel but government incentives were in place. Government funding has been reported as one of the main drivers for EMR adoption [18] and our findings support this. In the countries with lower adoption rates, government funding was present only in South Africa, but lacking in Brazil. However, several barriers towards EMR use were identified in these countries.

Different factors influenced the feasibility of data extraction for secondary uses, such as EMR adoption, quality of data, trends and incentives for EMR implementation and information governance procedures. Table 4 shows that it seems more feasible to extract EMR data for research in Italy, Saudi Arabia, Korea, Rep., Taiwan and UAE, but less feasible in Brazil and South Africa.

**Table 4:** Recommendations regarding the feasibility of data extraction from EMR for secondary uses

Countries	Feasible to extract data from EMR?	Factors influencing recommendation				
		EMR adoption	Quality of data	Implementation trends and incentives	Information governance procedures	Other
Italy	More feasible, optimal regions might include Abruzzo, Piemonte, Lazio, Lombardia and Trento.	High adoption, particularly in general physician clinics.	High fill rates. Already good linkage between EMR systems in general physician practices and hospitals.	Funding incentives.	Clear process. Could take a long time.	Existing research using EMR extracted data.
Saudi Arabia	More feasible, data from public sector.	High adoption in governmental facilities.	High fill rates. Comprehensive data available.	Increasing implementation. Future plans for unified EMR.	Clear process for public sector, but not for private sector. Could take a long time.	Health research oriented facilities exist.
Korea, Rep.	More feasible.	High adoption, particularly in general physician clinics and tertiary hospitals. Low fragmentation of providers in clinics, higher in hospitals.	High fill rates. Comprehensive data available. Consistency of EMR data.	Increasing implementation. Funding incentives.	Clear process. Moderately quick.	Existing research using EMR extracted data including diabetes research.
Taiwan	More feasible, optimal setting may be larger cities or institutions.	High adoption nationwide.	High fill rates. Comprehensive data available.	Increasing implementation. Funding incentives.	Clear process. Variable time.	Existing research using EMR extracted data.
UAE	More feasible, optimal setting in might include Health authority Abu Dhabi (HAAD) affiliated healthcare facilities (SEHA).	High adoption in general physician clinics and hospitals.	High fill rates. Comprehensive data available.	Increasing implementation. Different incentives in the public sector.	Clear process in SEHA facility. Moderately quick.	
Brazil	Less feasible	Overall low adoption, centered in a few hospitals and clinics. High fragmentation of providers.	Inconsistency of EMR data between sites.	Slowly increasing implementation. Government initiatives are poor and just beginning.	Clear process. Could take a long time.	Public systems are very difficult to access for research; clinic by clinic basis in the private sector.
South Africa	Less feasible, but when done an optimal setting may be major tertiary institutions in the Western Cape region or directly with the Ministry of Health.	Overall low adoption, higher adoption in private general physician clinics.	Available data are likely to be of modest quality and quantity.	Rapid increase. Attempts for interoperability.	No clear process. Takes a long time.	The use of EMR extracted data is very difficult.

Barriers towards EMR use have been extensively studied [4, 6, 20, 21, 29], though there remains limited evidence that can provide an understanding of the organisational context and process changes. The barriers found in our study were mostly in line with those previously reported in other countries. A systematic review on the costs and benefits of health information technology found that costs and perceived resistance by physicians were the main barriers towards EMR adoption [13]. In our study, costs were reported as a barrier, but resistance by physicians was not mentioned by our interviewees, which may have been because we interviewed stakeholders who knew about EMR in their setting and these stakeholders may have been less resistant towards EMR use.

Another review reported on concerns about patient privacy and legal barriers to the use of EMR [21]. Even though European countries have comprehensive national privacy laws and information commissioners, in our study concerns about data security were still mentioned in the European countries. If a study with data from EMR is undertaken at a single institution, approvals can be easily manageable. However, when a regional or national study is undertaken the approvals become hard to manage [7], which interviewees also reported in our study.

A limitation of the study is its commissioned nature, which meant that the included countries and the questionnaire were chosen by the client. This also meant that a purposive sample was included and that some countries (Mexico and Czech Republic) only had one interviewee. We used a rapid approach and asked participants to respond within a short period of time (two weeks) and thus the answers were self-reported when no interview could take place. However, this approach allowed us to share up-to-date and new insights into the information governance procedures and adoption and quality of EMR in a number of countries.

Another limitation is that this study only assessed three aspects of data quality: type of fields, fill rates and closed-ended fields. Other aspects of EMR systems, which vary on multiple dimensions, could not be assessed, including the details (clinical data, non-clinical data), data source, level of complexity, incorporation of other documents (digital images, scanned documents) timeframe (single occasion to complete health record) and extent of integration with other services [5]. Nevertheless, this study provides an important overview of the most relevant aspects of the quality of EMR for extracting data for research.

Future research in this area could explore other aspects of data quality and adoption. Both benefits, including data security, legibility, accessibility, completeness, comprehensiveness, efficiency, and risks such as paper persistence, patient disengagement, insecure data, increased time, and increased costs should be assessed [5]. Research is also needed on the views of patients and the public about data from their EMR being used in areas not directly related to providing them with clinical care [30, 31].

## Conclusions

This is the first international comparative study to shed light on the information governance procedures and adoption of EMR in several countries. We hope it will inform the discussions and development of policies that aim to accelerate adoption of EMR and seizing of the rich potential for the secondary uses of data arising from them. Data from EMR have considerable scope to improve the safety, quality and efficiency of healthcare; as well as being a valuable resource for research, particularly when linked to other data such as mortality records or genetic data. It is therefore important that countries work towards making these data more accessible for their secondary uses. At the same time confidentiality of the data should be ensured. Also any concerns that patients and the public might have about their data being used for purposes other than providing them with clinical care should be addressed. Last but not least, it is very important also to establish mechanisms for use of such data at national levels. They provide an invaluable source for policymakers, for measurement of disease burden and for planning of investments in healthcare as well as for pharmaceuticals to ensure safe and effective use of medications.

## Abbreviations

EMR, electronic medical records; HAAD, Health Authority of Abu Dhabi; HbA1c, glycated haemoglobin; SEHA, Abu Dhabi Health Services Company; UAE, United Arab Emirates

## Additional files

Additional file 1: Information Governance; summarized results. (PDF 567 kb) Additional file 2: Adoption of EMR and quality of their data; summarized results. (PDF 463 kb) Additional file 3: EMR adoption in the countries based on literature review; description. (PDF 183 kb) Additional file 4: Number of interviewees; table. (PDF 186 kb)
