# Supplementary material for: Feasibility of extracting data from electronic medical records for research: an international comparative study
Source: BMC Med Inform Decis Mak. 2016 Jul 13;16:90. doi: 10.1186/s12911-016-0332-1 (PMC4944506; doi:10.1186/s12911-016-0332-1)
Supplement: Additional file 1: — Information Governance; summarized results. (PDF 567 kb) [file 12911_2016_332_MOESM1_ESM.pdf]

Information governance

Information governance: Australia

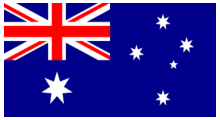

Insights	Summary
➢ Authorities who need to provide approval for EMR extraction and use of EMR data for research purpose	• Ethics board: ethics board of the health facility or regional human rights ethics committee<br>• Sites where data was collected: if data is extracted directly from the sites<br>• National, regional or local health authorities not required. Note that the Health Research Ethics Committee may be affiliated to the DOH<br>For private sector data additionally:<br>• Practice manager/business owner<br>• EMR system provider<br>• Individual patient consent is complex: Consent needed if data was extracted from private GP practices and when data is being collected in patient-identified form for research purposes, and later anonymised. Generally signed consent on a printed form is expected from the patient (or holder of their advanced healthcare directive authorisation). Consent must be given for each specific research "project"
➢ Process to obtain approval	For public sector data:<br>• Ethics approval from all universities and hospitals involved<br>• Agreement with sites<br>• Individual patient consent not required for federal government collected data, data kept for admin and audit purposes and anonymised data from state data linkage units<br>• Agreement with practice manager<br>• Business deal with EMR system provider<br>• Full disclosure of use of data and obtaining consent from all individuals
➢ Approximate time needed to obtain all approvals	• 6 months, however, the time depends on number of sites and source of the data. Each ethics committee may require a different adjustment to the protocol . As a rule add one month for each ethics committee.
➢ Ease of obtaining approval	• **Moderately easy**; process to obtain approval is straightforward but may take a long time.<br>Hurdles:<br>• Approval from multiple human research ethics committees<br>• De-identification or perturbation of data from private sector<br>• Cost of administration.
➢ Regional differences	There are different ethics committees in each region, same principles but different procedures.

Information governance: Austria

Insights	Summary
➢ Authorities who need to provide approval for EMR extraction and use of EMR data for research purpose	• Data of the EMRs are not yet meant to be used for secondary purposes, including research.<br>**Rationale:**<br>• Although high rate of IT equipped GPs in Austria, data is not routinely collected;<br>• EMR rollout is just beginning, many patients are opting out and many GPs still oppose it;<br>• No coding system is implemented in primary health care: reasons for contact or results after consultation/diagnoses is not coded but individually described;<br>• The data available so far are routine data produced used for remuneration purposes;<br>• Several different software systems are used;<br>• Companies are interested in collecting data themselves to sell them.<br><br>Authorities who would need to provide approval, **should this situation change**:<br>• Ethics boards: one for each of the 9 Austrian counties . Only required for scientific studies and publications<br>• National regional or local health authority: “Hauptverband der Sozialversicherungsträger” (roof organization for all 19 health insurance companies)<br>• Site where data was collected (GP or medical director, as the data owner and approval requirement depends on their interest)<br>• Individual patients at GPs office. Not required if agreed for data collection in medical context beforehand<br>• If the government would mandate data extraction, ethical approval would not be needed. However, dedicated laws would need to be passed beforehand, which is unlikely to happen.
➢ Process to obtain approval	• Not applicable, because EMRs cannot be used for research at the moment.
➢ Approximate time needed to obtain all approvals	• Results of a pilot study: 1 to 2 years.
➢ Ease of obtaining approval	• **Not feasible** to extract data for research purposes in Austria.<br>Hurdles:<br>• The medical association of Austria is strictly against the use of electronic health data for research purposes. Therefore, the situation in Austria is **very difficult**.<br>• Data protection legislation (cf. § 14 Abs. 2 GTelG 2012)<br>• Interest of the payers to get the results they want and not objective data.
➢ Regional differences	• Not applicable.

Information governance: Brazil

Insights	Summary
➢ Authorities who need to provide approval for EMR extraction and use of EMR data for research purpose	• **No formal government approval entity**<br>However, recommended route:<br>• Ethics board: Plataforma Brasil (a unified ethical board). National ethics committee - Comissão Nacional de Ética em Pesquisa (for clinical and most likely observational studies , particularly if sponsored by multinational laboratories), hospital ethics committees (for studies within hospitals).<br>• National, regional or local health authority: Municipal Secretary of Health, State Secretary of Health or the Ministry of Health, depending on who manages the EMR system.<br>• Individual patients: required in some cases, depending on evaluation by the ethics board. Individual approval is usually not required if data is anonymous.
➢ Process to obtain approval	• Ethics boards: submission of a detailed project proposal that is understandable by non-specialists.
➢ Approximate time needed to obtain all approvals	Total: 3-12 months, depending on organisation that holds the data:<br>• National ethics committee: 6-12 months;<br>• Hospital committee: 3-6 months.
➢ Ease of obtaining approval	• **Moderately easy**, depends on the organisation that holds the data.<br>• Approval depends on **setting** as each hospital, doctor or institution is responsible for the confidentiality.<br>• Important to prove that the data collection will have real benefit to the entity (private) or national healthcare policies (public).<br>• Transfer of data internationally is not a problem - some EMR companies already store data in data banks outside Brazil<br>Differences between public and private sector:<br>• Public: all systems owned by government and **very difficult to access** for studies;<br>• Private physicians office: **physician approval required**. Physicians might feel more comfortable in supplying data if the study approved in a larger ethical committee.
➢ Regional differences	• No information.

Information governance: China

Insights	Summary
➢ Authorities who need to provide approval for EMR extraction and use of EMR data for research purpose	• Ethics boards within the hospitals<br>• National and local health authorities e.g. Division of Medical Affairs and Division of Research: necessary if retrieving data from various provinces, or retrieving national data of various kinds such as public health, immunisation or epidemiology of emergency events<br>• Site where the data was collected: **director of the hospital**, hospital data centre<br>• Individual patients: In Hong Kong Special Administrative Region (HKSAR) consent needed for access to EHR. Not conclusive if required in mainland China .<br>• In HKSAR: the Office of the Privacy Commissioner for Personal Data, an independent statutory body, oversees enforcement of the Personal Data Privacy Ordinance (PDPO). Although not applicable for anonymised data, users should comply with requirements under the PDPO personal data handling. Suspected breaches will be investigated.
➢ Process to obtain approval	• Submission of Case Report Form (to ascertain potential harm to patients) and research proposal.<br>• However, there is no clear legal framework about data use rights.<br>Process for Hong Kong:<br>1. Application to the Secretary for Food and Health;<br>2. The Secretary for Food and Health may refer application to the Electronic Health Record Research Board;<br>3. The Board must consider several factors including ethical issues and public interest;<br>4. Applications for non-identifiable data are made to the Commissioner for Electronic Health Records (eHRC).
➢ Approximate time needed to obtain all approvals	• Less than 3 months
➢ Ease of obtaining approval	• **Relatively easy**, as there are procedures in place and the process is quick.<br>• The **director of the hospital** plays a key role in the approval process.<br>Hurdles:<br>• Organisation of the administration (no specific rules and regulations for data extraction)<br>• Potential technical problems at some sites due to own systems in hospitals, and concerns of data leakage in China.<br>• Law prohibiting transfer of non-anonymised EMR data outside HKSAR (section 33 of the PDPO), but not in force yet.<br>• Anonymised patient data can be used for research and preparing statistics relevant to public health or public safety if conditions for approval by the future Commissioner for the EHR under Division 3 of the eHRSS Bill are fulfilled.
➢ Regional differences	• Regional differences exist, e.g. Hong Kong has own authority dedicated to data protection.

Information governance: Czech Republic

Insights	Summary
➢Authorities who need to provide approval for EMR extraction and use of EMR data for research purpose	• Ethics board: at national level (driven by but separate entity from the Ministry), regional level (within large medical service providers) and faculty hospital level. Note that ethics boards are not strictly regulated by Czech legislation.<br>• National health authority: **Office for Personal Data Protection**<br>• Sites where the data was collected<br>• EMR system provider<br>• Individual patient consent not required for anonymised data: This is general rule arising from European and Czech data protection law
➢Process to obtain approval	• For ethics boards: description of the process and details of the planned data extraction.<br>• For Office for Personal Data Protection: consultation may be sufficient.<br>• For sites and EMR provider: agreement with or participation in the project.<br>• The data needs to be de-identified i.e. cannot be linked to an identified or identifiable data subject in their original form or following processing thereof<br>• Non-interventional clinical studies need to be registered with the State Institute of Drug Control (SUKL).
➢Approximate time needed to obtain all approvals	• Up to 3 or 6 months.
➢Ease of obtaining approval	• **Relatively easy**, because procedures are in place and the process is fairly quick.<br>• Data protection law does not prohibit the international transfer of anonymised<br>Hurdle:<br>• Personal data protection; the approval will depend on the structure of the data and links to personal IDs.
➢Regional differences	• There are a number of different ethics boards with different rules and regulations.

Information governance: India

Insights	Summary
➢Authorities who need to provide approval for EMR extraction and use of EMR data for research purpose	• Ethics board: Chair of an Independent Ethics Committee (e.g. within the university which is conducting the research), or, if looking at State EMR's then Indian Council of Medical Research's Ethics Committee will give approval<br>• Regional health authority: Chief Medical Officer of the district or the Regional Health Programme Controller<br>• Sites where the data was collected<br>• EMR system provider<br>• Individual patients: only for non-anonymised data
➢Process to obtain approval	1. Brief of research to be carried (detailed proposal stating aims, to avoid conflict of interest)<br>2. Ethics approval and permission for the State/National Health Department<br>• For care providers: data available "as required on demand" basis.<br>• In general, approval is **research setting specific** so if conducted at a university, the university's ethics committee must approve, the same applies for hospitals.
➢Approximate time needed to obtain all approvals	• Ideally 3 to 6 months, but it depends on the nature of the research to be conducted, ethical implications and the ready availability of data.
➢Ease of obtaining approval	• **Difficult** as it strongly depends on the region or institution.<br>Hurdles:<br>• Ignorance of authorities for this line of research: EMR are still in a very nascent stage;<br>• **Lack of uniformity** and scattered implementation/local rules and regulations;<br>• Getting access is a long and cumbersome task;<br>• Identification of correct EMRs is difficult;<br>• Variations among establishments especially if the research is national (causes longer process).
➢Regional differences	• There are large variations regarding the approval process in the different regions.

Information governance: Indonesia

Insights	Summary
➢ Authorities who need to provide approval for EMR extraction and use of EMR data for research purpose	• Hospital or university based ethical boards<br>• Local health authority<br>• Sites where the data was collected<br>• Individual patients
➢ Process to obtain approval	1. Physicians agree to participate in the study;<br>2. Approval from hospital manager;<br>3. Informed patient consent;<br>4. Submit the documents listed below to ethics committee:<br>- Ethical committee form and admission fee;<br>- Research protocol and licence application letter;<br>- Investigator's CV;<br>- Copy of information for patients.
➢ Approximate time needed to obtain all approvals	• 3 to 6 months, more likely around 3 months.<br>• Sometimes a revision of the research proposal is needed, which slows down the approval process.
➢ Ease of obtaining approval	• **Moderately easy.** The process to obtain approval is well-defined, but the lack of uniformity poses the main challenge.<br>Hurdles:<br>• Bureaucracy and involvement of different stakeholders;<br>• The EMR policy for Indonesia is not available until probably 2015. Each Ethics committee or health care provider has its own policy;<br>• EMR in Indonesia is limited to ambulatory settings. Inpatient information is still stored in paper based medical records combined with electronic based records.<br>• Research by foreigners requires local sponsor and partners
➢ Regional differences	• Differences in approval procedures as the Indonesian system is highly fragmented.

* EMR use in Indonesia is fragmented and just developing with no clear policy or procedure. Some of this information is drawn from processes for observational research and clinical trials

Information governance: Italy

Insights	Summary
➢Authorities who need to provide approval for EMR extraction and use of EMR data for research purpose	• Local ethics board: there is one ethics boards in each local health care authority, i.e. several hospitals may belong to the same authority and several local authorities are located in one region – there is no national health care authority involved.<br>• Local health authority<br>• Site where the data was collected: for hospital data would require approval from the Director and of the ethics committee<br>• EMR system provider: if data collected from individual GPs<br>• Patient consent: not necessary for anonymised data , but usual practice
➢Process to obtain approval	• The requirements depend on the **type of study**: retrospective (notification to the ethics board), prospective (need authorization from local ethics board – formal approval from local health authority. No standard procedure of document required etc. between local health authorities)<br>• Confidentiality according the Helsinki principles must be assured.<br>Special database for diabetes patients, developed by Italian Association of Medical Diabetologists (AMD):<br>• Information from 300 of 650 diabetes clinics with detailed high quality data and open field text. Most of these centres use the MyStar Connect software (formerly EuroTouch) managed by Meteda Sanofi. Approval from AMD is all that is required, i.e. the process is much quicker.
➢Approximate time needed to obtain all approvals	• 6 months to 1 year
➢Ease of obtaining approval	• **Moderately easy**, because it may take a long time to obtain approval.<br>Hurdles:<br>• Confidentiality must be assured.<br>• Reluctance to provide data: general attitude not to provide individual patient data, despite being anonymised, without consent form signed by patient authorising use of his/her personal data.<br>• Bureaucracy: time-consuming<br>• Difficult to obtain authorization for transfer of anonymous EMR data outside Italy, but could be possible.
➢Regional differences	• The local ethics boards are independent and therefore have own policies. The differences are immense.

Information governance: Mexico

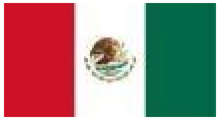

Insights	Summary
➢Authorities who need to provide approval for EMR extraction and use of EMR data for research purpose	• <u>Ethics Board:</u> depends on the chosen institution. Mexican Institute of Social Security (IMSS) has its own - IMSS National Research Commission and Ethics Committee.<br>• <u>Local health authority:</u> Mexican Institute of Social Security (IMSS) and the IMSS Division of Innovation and Development which has the centralized access to EMR from all IMSS family medicine clinics; or Institute for Social Security and Services for State Workers (ISSSTE).<br>• <u>Individual patients</u>: if data is non-anonymised, otherwise not
➢Process to obtain approval	1. Approval of ethical and research committees (e.g. IMSS National Research Commission and Ethics Committee) – requires submission of a detailed research protocol. IMSS requires submission by an IMSS researcher (international studies require collaboration with IMSS researcher(s).<br>2. For IMSS only: Approval of IMSS Division of Innovation and Development - requires being previously accepted by the IMSS National Research Commission and Ethics Committee.
➢Approximate time needed to obtain all approvals	• 6 months to 1 year (IMSS & ISSSTE)
➢Ease of obtaining approval	• **Moderately easy**, because it may take a long time to obtain approval.<br>Hurdles:<br>• Confidentiality: law to prohibit access to personal data (EMR included), so access will be limited;<br>• The private sector is not well organized; private doctors usually do not have EMR systems installed.
➢Regional differences	• No information

Information governance: Netherlands

Insights	Summary
➢ Authorities who need to provide approval for EMR extraction and use of EMR data for research purpose	• Ethics board: "Medisch-ethische Toetsings Commissie Mens Gebonden Onderzoek". In general, extraction of anonymous data is exempt from ethical approval. Up to research group to still notify the ethical boards, out of caution. If extracting from small populations, chance of identifying an individual might still be high.<br>• National health authority (at least for NIVEL data): approval is needed from national professional organisations of GPs that are represented in the Netherlands Institute for Health Services Research (NIVEL) primary care database governance structure<br>• Site where the data was collected: e.g. GP's<br>• Individual patient: informed consent. Officially this should not be required for anonymous data, but the scale and the relation of trust make this a prudent policy to avoid problems.
➢ Process to obtain approval	• Ethics board requires a number of conditions, including a study proposal, protection of patients against identification, informed consent, possibly of opting-out without consequences for further patient care and that results of the study are displayed in public domain.<br>• Site approval also based on study proposal and right of patients to opt-out. They will also assess the burden on patient care.<br>• A governance structure is in place, where professional organisations decide upon the use of the data for research projects.<br>• Notification of the Dutch Data protection Authority only needed if data is non-anonymised.
➢ Approximate time needed to obtain all approvals	• 3 to 6 months<br>• There is a difference between research of data that have been anonymised, and data that make it possible to identify the person. For both situations the procedure of informing is the same, but in case there is individual disclosure possible, the scrutiny is higher, the time involved longer.
➢ Ease of obtaining approval	• **Relatively easy**, because the process to obtain approval is straightforward.<br>• Anonymised data can be included in international studies and travel over the border<br>• Hurdle:<br>• Necessity of publication in the public domain.
➢ Regional differences	• Process is expected to be similar in different regions.

Information governance: Poland

Insights	Summary
➢Authorities who need to provide approval for EMR extraction and use of EMR data for research purpose	• Ethics board at regional level e.g. university or hospital: must be informed that a given study is performed including its purpose and goal<br>• Site where the data was collected: e.g. private health care provider - business conditions negotiation (CEO needs to approve, approval from ethics board not necessarily needed)<br>• EMR system provider: approval may be needed<br>• Individual patients: possible but not necessary if data are fully anonymized<br>• Inspector General for Personal Data Protection (GIODO): for non-anonymised data
➢Process to obtain approval	1. Documents required varies with ethics committees but in general : protocol and informed consent, document describing what the research is about, i.e. study design, methods of analysis and plans for publications of results. Data need to be anonymised in any case (public and private setting).<br>2. Agreements then need to be set up with the sites.<br>3. Contracts with EMR vendors may be required on a case by case basis.<br>Process in private health care provider LuxMed: (likely to be similar in competitor Medicover)<br>1. Detailed research proposal to be submitted to the Management board<br>2. Two supporting statements from members of the board who deal with clinical aspects of work.<br>3. Approval from the Medical Director, then co-signed by CEO.
➢Approximate time needed to obtain all approvals	• 3 to 6 months (closer to 6 than 3 months)
➢Ease of obtaining approval	• **Moderately easy**, because some obstacles have to be overcome.<br>• Slightly easier to obtain approval in private settings than in a public setting.<br>• International data transfer possible in both private and public setting. In private, final decision rests with ethics board. When scope of data is only selected disease or a few diagnoses , likely to transfer the data abroad, especially if owned by an international health care provider (e.g. BUPA owned LuxMed).<br>Hurdles:<br>• Obtaining trust, technical issues (internal IT specialist required), data security, lawyers;<br>• The most convenient set of data are raw data from EMR for further analysis.
➢Regional differences	• The procedures for obtaining approval are the same regardless of the region.

Information governance: Saudi Arabia

Insights	Summary
➢Authorities who need to provide approval for EMR extraction and use of EMR data for research purpose	• Ethics boards<br>• National, regional and local health authorities belonging to the ministry of health : National Committee of Medical & Bioethics or Research Committee at King Abdulaziz Medical City (KAMC) in Riyadh (not necessary for EMR extracted data)<br>• Site where the data was collected<br>• Provider and patient approval are not needed.
➢Process to obtain approval	• Approval from the hospital committees and the university conducting the research and requirements vary between sites<br>• Documents to be submitted for approval to the ethics board include: literature review, research question, ethical consideration of the study, and the planned variables to be extracted<br>• Private sector: There is no clear approval process, as EMR systems are not yet fully established.
➢Approximate time needed to obtain all approvals	• 6 months to 1 year
➢Ease of obtaining approval	**Moderately easy,** but hurdles have to be overcome, and strongly depends on the setting.<br>Hurdles:<br>• Lengthy procedure of approval, varies among hospitals (online approval vs. hardcopy);<br>• Limited experience in research nationwide;<br>• Cooperation of individuals involved and their understanding of research value;<br>• Lack of communication can occur between the ethics board (conveyed by its approval of the research) and data extractors regarding which variables were approved for extraction;<br>• Private sector: The data cannot be used for research as it is not fully developed and linked.
➢Regional differences	• **Ethics boards** are hospital specific and therefore approval is needed from each hospital where data is collected.

Information governance: South Africa

Insights	Summary
➢Authorities who need to provide approval for EMR extraction and use of EMR data for research purpose	• Ethics boards (institutional, provincial and national ethics committees.)<br>• National, regional or local health authority: not compulsory but may speed up the process<br>• Site where the data was collected<br>• The head of the health establishment concerned<br>• The owner of the data, whether this is a hospital or the EMR provider.<br>• Patient consent: always. However, depending on type of data, research environment, previous research and how questionnaire is set up, the research body might ask for individual consent or view it as already obtained in past.<br>• Depending how and where, consent required either from DoH (public), patients or private facilities (private), or both.
➢Process to obtain approval	• No identifiable personal information may be used for study, research or teaching unless patient has authorised the disclosure for that particular purpose.<br>• For case reports, photographs or other images to be published, consent must be given by patient whether identifiable or not.
➢Approximate time needed to obtain all approvals	• Depends on the interactions with the EMR provider and commitment of individuals.<br>• One senior academic in the EMR field had tried to obtain approval for 1 to 2 years – unsuccessfully.
➢Ease of obtaining approval	• **Difficult** due to lack of uniformity and length of the process.<br>• Partnerships with tertiary hospitals , especially those with their own ethics boards; or working with an organisation with an established ethics body linked to government (e.g. a local University) are recommended to speed up the process (3-6 months total according to a medic)<br>Hurdles:<br>• Obtaining consent and approval at multiple levels<br>• Finding the right research environment<br>• **Fragmented systems** and lack of interoperability<br>• **Delays** in getting a response<br>• Limited bandwidth and low levels of ICT<br>• High patent, utilization and license costs<br>• Poor staff literacy<br>• Individual autonomy of practice in private care may hinder data gathering in that sector
➢Regional differences	• Large variations in the process between the different regions.

Information governance: South Korea

Insights	Summary
➢Authorities who need to provide approval for EMR extraction and use of EMR data for research purpose	• Ethics boards: institutional review board (IRB) of the sites where the study is conducted.<br>• Site where the data was collected
➢Process to obtain approval	• Obtaining ethical approval for anonymised (de-identified) data from institutional review board of the sites.<br>• Obtaining consent of the sites is needed for EMR system providers.<br>• Data management staff usually extracts the data from the EMR and sends it to the investigators in an anonymous format.
➢Approximate time needed to obtain all approvals	• 3 to 6 months: depends on board you submit to and how fast you respond to their queries.<br>• Typically Research Ethics Committee (e.g. that at University of Pretoria) take up to 3 months.
➢Ease of obtaining approval	• **Relatively easy** process nationally, especially when concentrating on bigger hospitals.<br>Hurdles:<br>• Cost negotiation process would be the main hurdle for obtaining approval. Data has to be bought from local EMR system providers until this point.<br>• Korea has a strong privacy act and therefore it would be difficult to transfer EMR patient data internationally, even if anonymous<br>• Cannot extract from EMR without every institutes' IRB agreement.
➢Regional differences	• No information.

Information governance: Taiwan

Insights	Summary
➢Authorities who need to provide approval for EMR extraction and use of EMR data for research purpose	For National Health Insurance Research Database (NHRID) data:<br>• Ethics board: Institutional Review Board in each hospital or similar ethics board at the researcher's institution or approval from the National health research institutes ethics committee.<br>• Joint Institutional Review Board (JIRB, http://www.jirb.org.tw/) approval before conducting a multicentre trial in Taiwan may facilitate the process of getting approval from IRB of each site.<br>Non-NHRID data:<br>• If data is bought from EMR providers, they will take care of site and patient approval.
➢Process to obtain approval	Same process for both routes:<br>• Written informed consent can be waived if the data is de-identified.<br>• Chinese citizenship (NHIRD is available to domestic researchers only), privacy agreement, small processing fee<br>• Proposal/protocol stating what information is to be collected, for what purpose, how it would be shared and disposed of at the end.<br>• Dept. of Health should be informed which entity it is to acquire the data, and credibility of the entity.
➢Approximate time needed to obtain all approvals	• Varying information: 3 months to 6 years. Plans to reduce this to 15 days in the future.
➢Ease of obtaining approval	• **Relatively easy**, the procedure is quite straightforward.<br>Main hurdle:<br>• Approval from the Institutional Review Boards
➢Regional differences	• Coverage by the NHIRD is almost universal. Therefore, there are no major differences between regions.

Information governance: UAE

Insights	Summary
➢Authorities who need to provide approval for EMR extraction and use of EMR data for research purpose	• Ethics boards: within hospitals or universities where the data was collected, e.g. Al Ain medical district under the United Arab Emirates University (UAEU)<br>• SEHA (healthcare provider of the health authority of Abu Dhabi) ethics committee recently established (not always necessary for approval but preferred).<br>• Patient consent is not required.
➢Process to obtain approval	1. The facility must be licensed to conduct research by the health authority.<br>2. For each research conducted, an application form must be filled.<br>3. The study protocol is required for submission to the ethics committee at the hospital for approval.
➢Approximate time needed to obtain all approvals	• 3 to 6 months.
➢Ease of obtaining approval	• **Relatively easy** when focussing on a SEHA facility.<br>Hurdles:<br>• For a large numbers of patients' data, the server can get overwhelmed (not equipped for high quantity of data);<br>• Staff are not highly skilled on the extraction of EMR data;<br>• Might be time consuming.
➢Regional differences	• The regulations **vary** from hospital to hospital and can be emirate dependent.
