# Supplementary material for: Feasibility of extracting data from electronic medical records for research: an international comparative study
Source: BMC Med Inform Decis Mak. 2016 Jul 13;16:90. doi: 10.1186/s12911-016-0332-1 (PMC4944506; doi:10.1186/s12911-016-0332-1)
Supplement: Additional file 2: — Adoption of EMR and quality of their data; summarized results. (PDF 463 kb) [file 12911_2016_332_MOESM2_ESM.pdf]

Adoption of EMR and quality of their data

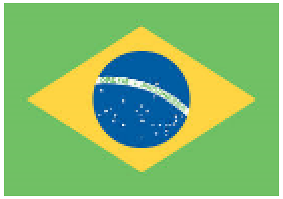

Brazil

Adoption of EMR and quality of their data

Treatment settings, EMR adoption and data quality

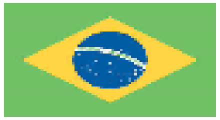

Insights	Summary
**Treatment setting**	
➢Typical treatment settings for type 2 diabetes patients initiating on second-line treatment	• All basic care outside hospitals.<br>• Public system = public clinics (GPs and specialists). Limited access to medication, usually low cost drugs given, dispensing often not recorded.<br>• Private system (25% of Brazilian population) = specialist physicians' office/clinic. This is where drug consumption is.
**EMR adoption**	
➢EMR adoption rate in the typical treatment setting	• Highly varied responses: GPs 5-40%, Specialists 5-50%, Hospitals 7-80%, Emergency units 50%. Difficult to capture as it depends on each physician and office.<br>• Low overall adoption<br>• Primary reason for reluctance to EMR and persistent paper culture: concerns among healthcare institutions regarding the security of patient data/concept of physicians owning their patient data and not wanting to forward this to other physicians.
**Overall quality and consistency of EMR data**	
➢Typical fields covered in the EMR system	• Depends on the type and structure of the system used by physician. This would be a clinic by clinic exercise.<br>• No electronic prescriptions.<br>• Both public and private hospitals (providing public services) have central database for claims data : only high-cost procedures and high-cost drugs dispensed.<br>• More data captured in public system as attended by different physicians each time (EMR more favourable)
➢Average fill rate (%)	• Difficult to capture. This would be a clinic by clinic exercise.
➢Fields with close-ended questions	• As physicians are protective of patient data, open fields may be more common than closed-ended. Some niche specialised systems exist with parameters used by the type of specialist, perhaps more likely to have close-ended questions.

Details of EMR systems

Insights	Summary	
	GPs	Hospitals
➢ Top EMR provider	MV Sistemas	MV Sistemas
➢ Geographic coverage and market share	Nationwide, 40-59% (>400 clients)	Nationwide, 40-59% (>400 clients - mainly hospitals)
➢ Top software options	MV 2000	• MV 2000
➢ Key fields captured and fill rates	• Patient information<br>• Patient vitals<br>• Diagnosis<br>• Prescription<br>• Procedure/test performed<br>• Lab test results<br>• Patient behavioural data	• Patient information<br>• Patient vitals<br>• Diagnosis<br>• Prescription<br>• Procedure/test performed<br>• Lab test results<br>• Patient behavioural data
➢ Ability to link clinical information from other sites	• No information available	

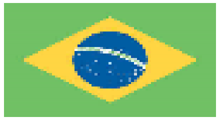

Future trend and incentives on EMR use

Insights	Summary
➢Overall trend on EMR implementation	• Trend is growing and expanding slowly<br>• All initiatives are confined to private market<br>• No large changes in regulations or government mandatory imposed policy that will make change happen faster.<br>• However, much interest from EMR vendors and accelerating activity expected in the next few years expected as Brazil is the third largest world market for EMR: with >200 million inhabitants, >7000 hospitals, >300,000 physicians, and a mixed public and private healthcare system.<br>• The EMR market earned revenues of US $145 million in 2012 and estimates to reach US $336 million in 2018 at a compound annual growth rate of 15 percent.
➢Incentives for EMR implementation	• Most important incentive is the necessity to improve services and coordination of care as public and private health sectors are stretched. Likely to follow other countries in EMR implementation.<br>• Incentives are sectorial, with independent motivations and initiatives.<br>• Local healthcare information technology (HC IT) market stage drives providers to offer EMR as a module pack within a hospital information system (HIS) solution.<br>• Very little national or regional incentives. In some states and some cities only.

Recommendation

Insights	Recommendation
Recommendation on Go/No-go for EMR extraction and rationale	**Do not proceed to EMR extraction:**<br>✓ Minimal uptake of EMR in Brazil, centered in a few hospitals and clinics;<br>✓ High fragmentation;<br>✓ Inconsistency of EMR data between sites;<br>✓ Government initiatives are poor and just beginning;<br>✓ Public systems very difficult to access for research, so would require proceeding on a clinic by clinic basis in the private market.

Italy

Adoption of EMR and quality of their data

Treatment settings, EMR adoption and data quality

Insights	Summary
**Treatment setting**	
➢Typical treatment settings for type 2 diabetes patients initiating on second-line treatment	• Specialists in hospitals plus GPs<br>• 99% public, ~40-50% diabetes patients attend specialists in public network of ~700 Diabetes Care Units (DCU) within hospital outpatient departments, usually referred by GPs. GPs are in charge of the transcription of the specialist prescription (Rx), for reimbursement of drugs. First prescription and change of therapy decided by diabetologist that sees patients every 3/6/12 months.
**EMR adoption**	
➢EMR adoption rate in the typical treatment setting	• GPs: 90%; however, level of use varies<br>• Hospitals/specialized clinics: 30-70% (varies across the regions)<br>• However, systems adopted in majority of diabetes clinics in all Italian regions
**Overall quality and consistency of EMR data**	
➢Typical fields covered in the EMR system	• Patient information<br>• Patient vitals<br>• Diagnosis<br>• Prescription<br>• Procedure/test performed<br>• Lab test results<br>• Patient behavioural data
➢Average fill rate (%)	• Average across providers: 80%; AMD database: very good (>90%) for treatment, process measures and intermediate outcomes, less so for final outcomes.
➢Fields with close-ended questions	CompuGroup software: Patient information (gender and age), diagnosis, prescription and procedure/test performed closed-ended. Free-text: lab test results<br>Italian Association of Medical Diabetologists (AMD) database: Open-ended in most fields

Details of EMR systems

Insights	Summary	
	GPs	Hospitals
➢ Top EMR providers	CompuGroup	Meteda Sanofi
➢ Geographic coverage and market share	Nationwide, 44% (22.000 GPs)	Nationwide; market share: 35% and used by 80%-100% of diabetologists in hospitals.
➢ Top software options	PROFIM 2000	MyStar Connect (new version to be released early 2015)
➢ Key fields captured and fill rates	• Patient information: 100%<br>• Patient vitals: 100%<br>• Diagnosis: 100%<br>• Prescription: 75%<br>• Procedure/test performed: 100%<br>• Lab test results: 100%<br>• Patient behavioural data: 60%	• Patient information (90%)<br>• Patient vitals (80%)<br>• Diagnosis (100%)<br>• Prescription (90%)<br>• Procedure/test performed (30-100%)<br>• Lab test results (70%)<br>• Patient behavioural data (20-70%)
➢ Ability to link clinical information from other sites	Yes: The software could be easily integrated with more questionnaires, if requested, for the recruited GPs.	The Meteda EMR system is already connected with the GPs EMR system in the regions Abruzzo, Piemonte and Lazio, due to regional/institutional regulations, set up with the aim of monitoring diabetic patients.

Details of EMR systems

Insights	Summary	
	GPs	Hospitals
➢ Top EMR providers	Cegedim	Not applicable
➢ Geographic coverage and market share	Nationwide, 40-59%	
➢ Top software options	Millewin or Millenium	
➢ Key fields captured and fill rates	• Patient information (100%)<br>• Patient vitals (100%)<br>• Diagnosis (100%)<br>• Prescription (75%)<br>• Procedure/test performed (100%)<br>• Lab test result (100%)<br>• Patient behavioural data (60%)	
➢ Ability to link clinical information from other sites	Yes, Cegdim is able to integrate patient level data collected by 600 GPs and patient level data collected by 100 specialist (by online questionnaires, not EMR) providing an integrated platform with longitudinal clinical patient information.	

Future trend and incentives on EMR use

Insights	Summary
➢Overall trend on EMR implementation	• The development of eHealth is mainly led by the implementation of the new National Health Information System NSIS, the E-Government Plan 2012 and the ongoing reform for federalism.<br>• Aiming for technological interoperability, unified terminology and communication structures between different health units<br>• More and more EMR's are used, however, the process is slow and unsystematic (especially for the part of hospital specialists).<br>Driving forces:<br>• Primary care physicians: economic benefits<br>• Specialists: continuous quality improvement<br>• Both: ease of consultation by multiple health providers.<br>• Promotion by scientific society and scientific publications
➢Incentives for EMR implementation	• The State annually shares out the National Healthcare Fund among the regions, according to the rules agreed in the State-Regions Conference. The Regions decide how much to allocate for eHealth activities.<br>• Examples of regional incentive: Lombardia with the project "cartella clinica elettronica" electronic health record; Trento "cartella clinica del cittadino«<br>• Incentives used to be the driving force at the primary care level, nowadays ago almost all GPs use EMR, however, the level of use varies.<br>• The software was provided free of charge to clinics.

Recommendation

Insights	Recommendation
Recommendation on Go/No-go for EMR extraction and rationale	**Proceed to EMR extraction:**<br>✓ High adoption of EMR systems<br>✓ High fill-in rates<br>✓ Already good linkage between EMR systemts in GP practices and hospitals<br>✓ Some incentives are in place<br><br>However:<br>➢ Process to obtain approval could be rather lengthy<br><br>Optimal regions might include Abruzzo, Piemonte and Lazio (as Meteda already links hospital to GP EMR systems, with the aim of monitoring diabetic patients) and Lombardia and Trento (as there are regional incentives for the use of EMR systems, indicating good coverage)

Saudi Arabia

Adoption of EMR and quality of their data

Treatment settings, EMR adoption and data quality

Insights	Summary
**Treatment setting**	
➢ Typical treatment settings for type 2 diabetes patients initiating on second-line treatment	• Mainly specialists and consultants in clinics or hospitals (e.g. university hospitals, military hospitals, National Guard hospitals and private hospitals).<br>• 59.5% of the care is provided by Ministry of Health facilities. Here more specialists are in hospitals (70%) than clinics (30%).<br>• GPs can initiate second line oral therapies, but this only applies to 2-3% of cases.
**EMR adoption**	
➢ EMR adoption rate in the typical treatment setting	• 20-30% of hospitals have EMR systems installed<br>• 100% in governmental facilities<br>• Highest adoption on the Electronic Medical Record Adoption Model (EMRAM) so far was achieved by the King Faisal Specialist Hospital & Research Centre (KFSH&RC) in Riyadh and Jeddah (level 6 out of 7).
**Overall quality and consistency of EMR data**	
➢ Typical fields covered in the EMR system	• Patient information<br>• Patient vitals<br>• Diagnosis<br>• Prescription<br>• Procedure/test performed: blood and urinary test<br>• Lab test results<br>• Patient behavioural data<br>• Hospitalisation
➢ Average fill rate (%)	• Almost 100% in KFSH&RC (mandatory by the system)
➢ Fields with close-ended questions	• Diagnosis, prescriptions, lab results

Details of EMR systems

Insights	Summary	
	GPs	Hospitals
➢ Top EMR providers	EMR systems are not yet established at primary health care centres for GPs, unless the GP is based within a hospital.	Medicaplus
➢ Geographic coverage and market share		Only in big cities and certain regions like Dammam, Jubail area. 20-39%
➢ Top software options		Medicaplus
➢ Key fields captured and fill rates		• Patient information (100%)<br>• Prescription (90%)<br>• Procedure/test performed: blood and urinary test (100%)<br>• Lab test results (100%)
➢ Ability to link clinical information from other sites		Yes, but only for prescription, procedure/test performed and lab test results

Details of EMR systems

Insights	Summary	
	GPs	Hospitals
➢ Top EMR providers	Not applicable	Cerner
➢ Geographic coverage and market share		Nationwide, including main cities of Jeddah and Riyadh
➢ Top software options		• Cerner
➢ Key fields captured and fill rates		• Patient information (100%)<br>• Patient vitals (not all, on average: 40%)<br>• Diagnosis (50-60%)<br>• Prescription (90%)<br>• Procedure/test performed (100%)<br>• Lab test results (100%)<br>• Patient behavioural data: only smoking<br>• Hospitalization
➢ Ability to link clinical information from other sites		Yes, for all of the abovementioned fields except from patient behavioural data and hospitalization

Future trend and incentives on EMR use

Insights	Summary
➢Overall trend on EMR implementation	• Some hospitals hesitate to offer remote access to EMR because of concerns about legal liability and privacy issues.<br>• However, overall general interest with huge investments in healthcare expected to reach >40 billion in 2017.<br>Driving forces:<br>• Quality improvement
➢Incentives for EMR implementation	• There are no national or regional incentives around installing EMR systems.<br>• The Ministry of Health has taken initiatives to enhance EMR adoption in healthcare settings in 2008 and 2010 and initiated a project to automate and start with 30 hospitals in different regions of the country including a unified electronic medical record in 2008. The future plan is for unified EMR system that allows physicians to access EMR data for patients treated at another site, however for the time being this is not possible.

Recommendation

Insights	Recommendation
Recommendation on Go/No-go for EMR extraction and rationale	**Proceed to EMR extraction:**<br>✓ High adoption, 100% in governmental facilities<br>✓ Comprehensive data available with high fill rates.<br>✓ Future plans for unified EMR<br>✓ Health research oriented facility exists<br><br>As major health providers are in the top 2 cities, the optimal setting for EMR based research in Saudi Arabia would be a governmental hospital setting especially King Faisal Specialist Hospital & Research Centre (KFSH&RC) in Riyadh and Jeddah.

South Africa

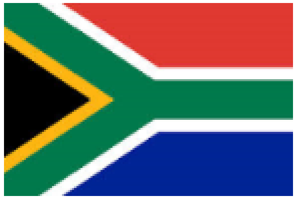

Adoption of EMR and quality of their data

Treatment settings, EMR adoption and data quality

Insights	Summary
**Treatment setting**	
➢Typical treatment settings for type 2 diabetes patients initiating on second-line treatment	• Private setting: GPs<br>• Public setting: District (secondary) or tertiary hospitals
**EMR adoption**	
➢EMR adoption rate in the typical treatment setting	• GPs: 100%; particularly in the private sector some form of electronic hospital management software (PCOE) is used<br>• For Hospitals: highest adoption rate in Western Cape (3 public tertiary hospitals and 1 clinic within the University of Pretoria use EMR)<br>• In general: The "paper culture" is still very much in force in both the private and public sector.
**Overall quality and consistency of EMR data**	
➢Typical fields covered in the EMR system	• Patient vitals<br>• Prescription<br>• Diagnosis<br>• Lab test results<br>• Procedure/test performed
➢Average fill rate (%)	• Within the Tygerberg hospital full medical history and data from each episode is stored .<br>• One public clinic reported 100%, however its one of only a few clinics in the public sector that uses EMR
➢Fields with close-ended questions	• Patient vitals, Prescription and Lab test results (for other fields: no information available)

Details of EMR systems

Insights	Summary	
	GPs	Hospitals
➢ Top EMR providers	Synaxon	VP Health
➢ Geographic coverage and market share	Nationwide, 80%	No information available
➢ Top software options	Synaxon	VEMR
➢ Key fields captured and fill rates	• Patient information (100%)<br>• Patient vitals<br>• Diagnosis<br>• Prescription<br>• Procedure/test performed<br>• Lab test results<br>• Patient behavioural data	• Procedure/test performed<br>• Lab test results
➢ Ability to link clinical information from other sites	Yes	No information available

Details of EMR systems

Insights	Summary	
	GPs	Hospitals
➢ Top EMR providers	Med-e-Mass	Not applicable
➢ Geographic coverage and market share	Nationwide, 60%	
➢ Top software options	HealthOne (Connect)	
➢ Key fields captured and fill rates	• Patient vitals<br>• Prescription<br>• Diagnosis<br>• Lab test results	
➢ Ability to link clinical information from other sites	Connect allows you to create, store and share patient records on an online platform.	

Future trend and incentives on EMR use

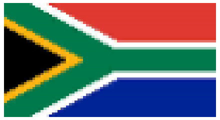

Insights	Summary
➢Overall trend on EMR implementation	• The department of health proposed a National eHealth strategy for 2012-2017.<br>• Rapid increase, especially in the private sector as EMR systems facilitate the reimbursements from the medical aid.<br>• Since 1994 each region has been involved in implementation and procurement of all health information systems. Data shows greatest success has been in the West cape region.
➢Incentives for EMR implementation	• As part of the National Health Insurance program there are attempts to ensure interoperability of health records at the National level. However, standards are yet to be agreed on and there is a lot of political interference.

Recommendation

Insights	Recommendation
Recommendation on Go/No-go for EMR extraction and rationale	**Do not proceed with EMR extraction:**<br>✓ The use of EMR extracted data is very difficult.<br>✓ For any planned study, it is best to partner with academic/researcher in the major tertiary institutions in the Western Cape region or directly with the Ministry of Health.<br>✓ Available data are likely to be of modest quality and quantity.

South Korea

Adoption of EMR and quality of their data

Treatment settings, EMR adoption and data quality

Insights	Summary
**Treatment setting**	
➢Typical treatment settings for type 2 diabetes patients initiating on second-line treatment	• General practitioners and specialist clinics both in primary care (41.5%) and hospital care<br>• Hospitals
**EMR adoption**	
➢EMR adoption rate in the typical treatment setting	• 65% for general practitioners and specialist clinics (2005 statistic), however believed to currently be ~90%<br>• 60% for hospitals:<br>- 77% for tertiary hospitals<br>- 40% for smaller hospitals
**Overall quality and consistency of EMR data**	
➢Typical fields covered in the EMR system	• Patient information<br>• Patient vitals<br>• Diagnosis<br>• Prescription<br>• Procedure/test performed<br>• Lab test result<br>• All, except behavioural data.
➢Average fill rate (%)	• 95%
➢Fields with close-ended questions	• All of the above mentioned except procedure/test performed (free text).

Details of EMR systems

Insights	Summary	
	Clinics	Hospitals
➢ Top EMR providers	UBCare	NGTech
➢ Geographic coverage and market share	Nationwide, 40-59%	Nationwide, less than 20%
➢ Top software options	Usarang	Mediplus EMR
➢ Key fields captured and fill rates	• Patient information<br>• Patient vitals<br>• Diagnosis<br>• Prescription<br>• Procedure/test performed<br>• Lab test result	• Patient information<br>• Patient vitals<br>• Diagnosis<br>• Prescription<br>• Procedure/test performed<br>• Lab test result
➢ Ability to link clinical information from other sites		No

Details of EMR systems

Insights	Summary	
	Clinics	Hospitals
➢ Top EMR providers	Not applicable	Choongwae Information Technology
➢ Geographic coverage and market share		Nationwide, less than 20%
➢ Top software options		CI OCS/EMR V.7
➢ Key fields captured **and fill rates**		• Patient information<br>• Patient vitals<br>• Diagnosis<br>• Prescription<br>• Procedure/test performed<br>• Lab test result
➢ Ability to link clinical information from other sites		No information available

Details of EMR systems

Insights	Summary	
	Clinics	Hospitals
➢ Top EMR providers	Not applicable	ezCaretech
➢ Geographic coverage and market share		Nationwide, less than 20%
➢ Top software options		ezCare-EMR
➢ Key fields captured and fill rates		• Patient information<br>• Patient vitals<br>• Diagnosis<br>• Prescription<br>• Procedure/test performed<br>• Lab test result
➢ Ability to link clinical information from other sites		No information available

Future trend and incentives on EMR use

Insights	Summary
➢Overall trend on EMR implementation	• Increasing implementation<br>• As there are so many different EMR systems, physician are still trying to unite EMRs. Currently most physicians have to make and print the summary about patient's records for transferring patients.<br>Driving forces are:<br>• Better patient and record management<br>• Seeking efficiencies, reducing standby time of patients, preventing record entry errors – competition among the hospitals<br>• Facilitating Electronic Data Interchange reimbursement claims.
➢Incentives for EMR implementation	• Yes, the **U health** scheme funds pilot projects around electronic health.

Recommendation

Insights	Recommendation
Recommendation on Go/No-go for EMR extraction and rationale	**Proceed to EMR extraction:**<br>✓ High uptake of EMRs, particularly in clinics and tertiary hospitals.<br>✓ Low fragmentation EMR sytem providers in clinics, but higher fragmentation in hospitals.<br>✓ Consistency of EMR data.<br>✓ Existing high quality research using EMR including diabetes research.<br>✓ Regulations are clearly defined and approvals are expected to be obtained moderately quickly.<br><br>A focus on clinics and larger tertiary centres with a large pool of patients and experience conducting research using EMR extracted data might be useful.

Taiwan

Adoption of EMR and quality of their data

Treatment settings, EMR adoption and data quality

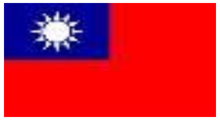

Insights	Summary
**Treatment setting**	
➢Typical treatment settings for type 2 diabetes patients initiating on second-line treatment	• GPs (40%) and hospitals (60%), also specialized clinics<br>• Main treatment setting is hospitals, where most physicians specialized in endocrine and metabolic conditions work. However, some specialists may open their own clinic.
**EMR adoption**	
➢EMR adoption rate in the typical treatment setting	• Medical centres: 100%. Treatment setting largest in relative size. Usually have >1000 beds.<br>• Regional hospitals: 67.5%<br>• District hospitals: 29.9%. Treatment setting smallest in relative size.
**Overall quality and consistency of EMR data**	
➢Typical fields covered in the EMR system	• Patient information<br>• Patient vitals<br>• Diagnosis<br>• Prescription<br>• Lab test results (hospital EMRs only, NOT required in NHIRD)<br>• Procedure/test performed<br>• Hospitalization
➢Average fill rate (%)	• 100%
➢Fields with close-ended questions	• None: all are free text questions

Details of EMR systems

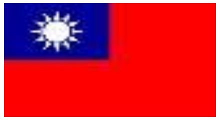

Insights	Summary	
	GPs	Hospitals
➢ Top EMR providers	All hospitals and GPs in both public and private sector use a state developed EMR that links to the NHIRD.	Government owned NHIRD
➢ Geographic coverage and market share		Nationwide, 80-100%
➢ Top software options		Developed by the government. Hospitals and GPs can create their own interphase and make modifications but they must all use the NHID codes and follow the HL7 CDA format.
➢ Key fields captured and fill rates		There are about 17 fields in the NHIRD, covering demographics (gender and DOB), diagnosis, medication, test requested but no lab test results. However, several test results are reported to NHI administration for patients enrolled in pay-for-performances program for diabetes care such as HbA1c, but this information is not yet open to public.
➢ Ability to link clinical information from other sites		Yes. Currently available to domestic researchers only but international cooperation can be performed via participation of local researchers. Transferring “raw data” overseas not allowed currently. However, researchers could raise the request with a purpose of international comparison. NHIRD does not welcome applications for commercial purpose.

Future trend and incentives on EMR use

Insights	Summary
➢Overall trend on EMR implementation	• The Department of Health started EMR implementation project in 2008, and since 2010 accelerated EMR implementation in hospitals. The country is setting up a nation-wide EMR exchange centre currently.<br>• It is planned that all 500 hospitals and 20,000 clinics in Taiwan will have EMR system implemented by 2016.<br>Driving forces:<br>• Government policy<br>• Cost saving (by reducing redundant examinations/ tests and speed up diagnosis)<br>• Improve healthcare quality (better clinical decisions based on previous patient treatment history)
➢Incentives for EMR implementation	• Under the accelerating EMR adoption program subsidies were provided to practices.<br>• There really is no need for private EMR vendors in Taiwan because the government has the largest share with almost universal coverage.

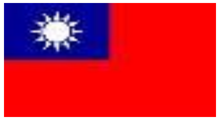

Recommendation

Insights	Recommendation
Recommendation on Go/No-go for EMR extraction and rationale	**Proceed to EMR extraction:**<br>✓ High adoption of EMR all over the country<br>✓ Comprehensive data available<br>✓ Straightforward process to obtain approval for data extraction<br>✓ Existing body of research using EMR data<br>✓ Focus on larger cities or institutions is recommended.

UAE

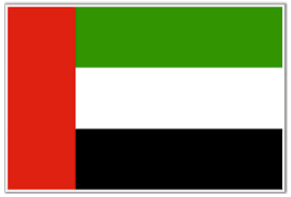

Adoption of EMR and quality of their data

Treatment settings, EMR adoption and data quality

Insights	Summary
**Treatment setting**	
➢Typical treatment settings for type 2 diabetes patients initiating on second-line treatment	• Specialists and consultants in clinics or hospitals. Roughly equal split of specialists are in clinics (~45%) and hospitals (~55%).<br>• 10 % GP based
**EMR adoption**	
➢EMR adoption rate in the typical treatment setting	• Almost universal in hospitals and clinics. In Abu Dhabi it is 100% and in other emirates also almost 100%.<br>• 100% in the Health authority Abu Dhabi (HAAD) affiliated healthcare facilities (SEHA)<br>• Al Ain have a unified EMR system (Malaffi) which service over 1.7 Million patients (representing 18% of the total population of the UAE)<br>• Private facilities are also mandated to use EMR so all use it with some using the minimum modules.
**Overall quality and consistency of EMR data**	
➢Typical fields covered in the EMR system	• Patient information<br>• Patient vitals<br>• Diagnosis<br>• Prescription<br>• Procedure/test performed<br>• Lab test results<br>• Hospitalization
➢Average fill rate (%)	• 100% (mandatory by the system)
➢Fields with close-ended questions	• Lifestyle questions such as smoking status, exercise and alcohol consumption.

Details of EMR systems

Insights	Summary	
	GPs	Hospitals
➢ Top EMR providers	Cerner	Cerner
➢ Geographic coverage and market share	Nationwide, 100% for SEHA	Nationwide, 100% for SEHA
➢ Top software options	Cerner	Cerner
➢ Key fields captured and fill rates	• Patient information (100%)<br>• Patient vitals [heart rate and blood pressure not always, no waist circumference] (100%)<br>• Diagnosis (100%)<br>• Prescription (100%)<br>• Procedure/test performed (100%)<br>• Lab test results (100%)<br>• Hospitalization: whether or not the patient is hospitalized (100%)	• Patient information (100%)<br>• Patient vitals [heart rate and blood pressure not always, no waist circumference] (100%)<br>• Diagnosis (100%)<br>• Prescription (100%)<br>• Procedure/test performed (100%)<br>• Lab test results (100%)<br>• Hospitalization: whether or not the patient is hospitalized (100%)
➢ Ability to link clinical information from other sites	Yes, if the GP is based within the hospital.	Yes, it is viewable from other sights such as Tawam hospital and Alain hospital shared EMR files.

Future trend and incentives on EMR use

Insights	Summary
➢Overall trend on EMR implementation	• Increasing trend towards paperless health records.<br>Driving forces:<br>• Innovation<br>• The desire to be original and leading in the EMR field<br>• Support of a well-trained team
➢Incentives for EMR implementation	• Yes, governmental incentives exist in the public sector, but not in the private one<br>• Examples of incentives include:<br>1. Exempting facilities with an approved inventory of an EMR from using controlled medication pads, which are time consuming and put the prescribers and hospitals at risk of investigation and license revocation<br>2. The inspection of facilities with an EMR is more lenient than the ones without.

Recommendation

Insights	Recommendation
Recommendation on Go/No-go for EMR extraction and rationale	**Proceed with EMR extraction:**<br>✓ Almost universal coverage in clinics and hospitals<br>✓ Comprehensive data available: EMR provider Cerner captures all fields with 100% fill rates.<br>✓ Straightforward process to obtain approval for data extraction, within a SEHA facility<br>✓ Incentives in the public sector<br><br>• Optimal setting for EMR data extraction in the UAE would be in a SEHA facility, mainly in Al Ain hospital in Al Ain and Sheik Khalifa medical city in Abu Dhabi due to previous EMR data extracted research and more experience.<br>• The Imperial College London Diabetes Medical Centre in Abu Dhabi and Al Ain is a private licensed research facility with an excellent EMR adoption rate.<br>• Regarding other emirates, the university of Sharjah hospital has an almost 100% EMR adoption and they are research oriented.
