# Supplementary material for: Feasibility of extracting data from electronic medical records for research: an international comparative study
Source: BMC Med Inform Decis Mak. 2016 Jul 13;16:90. doi: 10.1186/s12911-016-0332-1 (PMC4944506; doi:10.1186/s12911-016-0332-1)
Supplement: Additional file 3: — EMR adoption in the countries based on literature review; description. (PDF 183 kb) [file 12911_2016_332_MOESM3_ESM.pdf]

Additional File 3: EMR adoption in the countries based on literature search; description; PDF

The Australian Government has implemented EMR in the National Health Service since 2002 with the aim to integrate EMR systems across the country's eight regions [18]. In 2012, personal EMR were introduced, which allow patients to access and post information through a web portal [34]. In Korea, Rep., the Ministry of Health, Welfare and Family Affairs introduced a 5-year plan for implementation of interoperable EMR in 2005 [35]. In 2009, most private providers had adopted some form of EMR in Korea, Rep. [36]. In Taiwan, the Ministry of Health and Welfare has been promoting the use of EMR by general physicians since 2000 and introduced programs that aimed for up to 70% nationwide EMR adoption by 2012 [37].

In 2007, the Federal Health Commission in Austria recommended standards for EMR ("ELGA" or Elektronische Gesundheitsakte), and, in 2009, EMR systems were implemented across the country [38]. In the Czech Republic, efforts for EMR were initiated in 2003 and about 20% of the population and 1/3 of healthcare institutions were using an EMR by 2009 [39]. In Italy, the Ministry of Health and the Ministry for Public Administration and Innovation initiated efforts for EMR implementation in 2002. The implementation started at regional level in 2009 and was followed by plans to make basic patient summaries part of a national EMR by 2012 [40]. In the Netherlands, most general physicians, pharmacies and hospitals use EMR systems [41]. The deployment of a national infrastructure for EMR started in 2006 with the aim of full uptake, routine use and full patient access after 2009 [42]. In Poland, the National Health Fund began implementation of EMR in 2007, mainly for

cost control. An eHealth plan for 2010-2015 was set out, but lacked standards, dedicated healthcare solutions and a plan for data protection [43].

In Saudi Arabia, implementation of EMR slowly moved forward in the public sector, though information systems were not connected to each other or to private healthcare facilities [44]. The Saudi Arabian government introduced an eHealth plan with dedicated funding for the period 2008-2011, but a study showed adoption of EMR of only about 16% in an Eastern Province in 2010 [45]. The United Arab Emirates Health Authority of Abu-Dhabi (HAAD) implemented an US EMR system (Cerner) in Abu Dhabi and Al Ain in 2008. Despite initial difficulties with the EMR implementation, physicians demonstrated positive experience with the system [29].

The Indian government made recommendations for national EMR in 2007 and many hospitals had limited local EMR implementation in 2009 [36]. The Indian Ministry of Health & Family Welfare set out standards for EMR in 2013 [46]. In addition, the Indonesian government developed standards for the implementation of EMR mainly in hospitals in 2006 [47]. In 2013, only a few hospitals, mostly international hospitals, had implemented an EMR in Indonesia [48]. In China, some hospitals introduced EMR without going paperless in 2006 [47]. The Chinese government initiated a 5-year EMR strategy in 2009 [49]. South Africa published an eHealth strategy for 2012-2017 which aimed to implement a national EMR system [50]. Brazil introduced a foundation for EMR in 2000, but still lacked direction for a national program in 2009 [36]. Finally, EMR systems were introduced in Mexico by the Mexican Institute of Social Security (IMSS) in 2003 [30].
