# Supplementary material for: Feasibility of extracting data from electronic medical records for research: an international comparative study
Source: BMC Med Inform Decis Mak. 2016 Jul 13;16:90. doi: 10.1186/s12911-016-0332-1 (PMC4944506; doi:10.1186/s12911-016-0332-1)
Supplement: Additional file 4: — Number of interviewees; table. (PDF 186 kb) [file 12911_2016_332_MOESM4_ESM.pdf]

Additional File 4: Number of interviewees; table; PDF

Country	Total number of interviewees	Physician	Academic	Information commissioner	EMR provider
Australia	3	1	1	1	-
Austria	5	1	1	1	2
Brazil*	3	1	1	-	1
China	6	3	2	1	-
Czech Republic	1	-	1	-	-
India	5	3	2	-	-
Indonesia	2	-	1	1	-
Italy*	6	3	2	-	1
Mexico	1	-	1	-	-
The Netherlands	2	-	2	-	-
Poland	3	-	2	-	1
South Africa*	3	1	1	-	1
Saudi Arabia*	4	3	1	-	-
Korea, Rep.*	2	-	2	-	-
Taiwan*	6	3	2	-	1
UAE*	7	6	1	-	-
All countries	59	25	23	4	7

*Seven countries for which the extent of EMR adoption and the quality and consistency of EMR data was assessed
